# Sex-Determining Region Y Controls the Effects of Fetal Alcohol Exposure on Proopiomelanocortin Gene Expression

**DOI:** 10.3389/fnins.2021.608102

**Published:** 2021-03-16

**Authors:** Omkaram Gangisetty, Edward A. Mead, Dipak K. Sarkar

**Affiliations:** Rutgers Endocrine Research Program, Department of Animal Sciences, Rutgers University, New Brunswick, NJ, United States

**Keywords:** fetal alcohol exposure, SRY, POMC, epigenetic, transcriptional regulation

## Abstract

Fetal alcohol exposure (FAE) causes various neurodevelopmental deficits in offspring, including reduced expression of the stress regulatory proopiomelanocortin (*Pomc*) gene and an elevated stress response for multiple generations via the male germline. Male germline-specific effects of FAE on the *Pomc* gene raises the question if the sex-determining region Y (SRY) may have a role in regulating *Pomc* gene expression. Using a transgenerational model of FAE in Fischer 344 rats, we determined the role of SRY in the regulation of the *Pomc* gene. FAEs, like on the *Pomc* gene, reduced *Sry* gene expression in sperm and the mediobasal hypothalamus (MBH) in male adult offspring. Fetal alcohol-induced inhibition of *Sry* gene expression was associated with increased *Sry* promoter DNA methylation. Additionally, fetal alcohol effects on the *Sry* gene persisted for three generations in the male germline but not in the female germline. *Sry* gene knockdown reduced the *Pomc* gene expression. *Sry* recruitment onto the *Pomc* promoter was found to be reduced in the hypothalamus of fetal alcohol-exposed rats compared to control rats. *Pomc* promoter luciferase activity was increased following *Sry* overexpression. A site-directed mutagenesis study revealed that *SRY* binding sites are required for *POMC* promoter transcription activity. Overall, these findings suggest that SRY plays a stimulatory role in the regulation of *Pomc* gene expression and may potentially contribute to the fetal alcohol-induced changes in the level of *Pomc* gene expression for multiple generations.

## Introduction

Fetal alcohol exposure (FAE) has been shown to elevate neuroendocrine response of the hypothalamic-pituitary-adrenal (HPA) axis to stress (Ogilvie and Rivier, 1997; Hellemans et al., 2010; Bakoyiannis et al., 2014; Sarkar et al., 2019), partly due to a lack of the inhibitory control of the proopiomelanocortin (POMC)-derived peptide in the hypothalamus (Sarkar et al., 2007; Wynne and Sarkar, 2013). Additionally, it has been shown that replenishment of POMC neurons by neuronal transplants in the hypothalamus prevents stress hyperresponse in fetal alcohol-exposed rats (Boyadjieva et al., 2009), suggesting that FAE suppresses the level of POMC to alter the function of the HPA axis. The FAE effect on POMC involves epigenetic modification of *Pomc* DNA methylation via recruitment of MeCP2 on *Pomc* promoter (Govorko et al., 2012; Gangisetty et al., 2014). Interestingly, FAE-induced changes in *Pomc* promoter DNA methylation bypasses epigenetic reprogramming during embryonic development and are transmitted for multiple generations only through the male—but not female germline (Govorko et al., 2012; Sarkar, 2016), suggesting the possibility of involvement of a male-specific chromosome/gene which overcomes developmental editing and regulates *Pomc* gene expression. Paternal transmission of the Y chromosome can trigger testicular differentiation from bipotential gonads in mammalian embryos. The non-pairing region of the Y chromosome (Y^*NPAR*^) is transmitted from father to sons (Eyre-Walker, 1993) and has been proposed to bypass developmental molecular programming and therefore participates in the male-specific transgenerational transfer of the epigenetic mark (Li et al., 2002). The sex-determining region Y (SRY) is one of the functional genes involved in male reproduction that is mapped on Y^*NPAR*^. It has been proposed that β-endorphin, a functional peptide of POMC, is regulated by Y^*NPAR*^ that comprises SRY (Botbol et al., 2011). SRY is a transcription factor involved in regulating several genes in male sex determination (Sekido and Lovell-Badge, 2008; Bhandari et al., 2011). SRY also regulates endocrine response to stress (Dickey et al., 2012). SRY binding sites were identified to be altered transgenerationally in the Sertoli cell of the testis in vinclozolin-treated male offspring (Skinner et al., 2015). We hypothesize that *S*RY may have a role in regulation of *Pomc* gene expression and may contribute to the transgenerational effects of FAE on *Pomc*. We determined FAE effects on *Pomc* and *Sry* expression in the mediobasal hypothalamus (MBH) and sperm. We also conducted *Sry* gene knockdown experiments *in vitro* and *in vivo* and determined its effect on *Pomc* expression. Additionally, SRY recruitment onto the *Pomc* promoter was assessed in MBH of FAE rats. Furthermore, we determined *SRY* binding motifs on *Pomc* promoter and evaluated SRY regulation through its binding sites using promoter luciferase activity. The results of these studies are presented here.

## Materials and Methods

### Animals

All rat studies were performed with approved protocols in compliance with the Association for the Assessment and Accreditation of Laboratory Animal Care and Rutgers University. Fisher 344 strain rats were obtained from Harlan Laboratories (Indianapolis, IN) and housed in a controlled condition at a constant temperature of 22°C and 12-h light/dark cycles throughout the study. These rats were bred in our animal facility and used for this study. On gestational day (GD) 7 through 21, rats were fed rat chow *ad libitum* (AD), a liquid diet containing ethanol (AF; 1.7–5.0% v/v from GD7-10 and 6.7% v/v from GD11-21; Bioserve, Inc., Frenchtown, NJ), or pair-fed (PF; Bioserve) an isocaloric liquid control diet (with alcohol calories replaced by maltose-dextrin). Previous studies have shown that the peak blood ethanol concentration is achieved in the range of 120–150 mg/dl in pregnant dams fed with this ethanol-containing liquid diet (Miller, 1992). AF and PF litters were cross-fostered, and the litter size was maintained at 8 pups/dam. Only one pup from each litter was used in an experimental measure. The male offspring animals were used for the study and were sacrificed at the age of 2–3 months. Transgenerational studies were conducted by breeding AF, PF, or AD rats with control animals of the opposite gender to produce two germlines. The male germline (AFM or PFM) was generated by breeding male (AF or PF) rats and their male offspring with control (AD) females, and the female germline (AFF or PFF) was generated by breeding female (AF or PF) rats and their female offspring with control (AD) males. All rats were sacrificed at 60–90 days after birth for further experimentation. MBH was excised from the whole brain for experiments. Caudal epididymis was collected from male rats in a small petri dish containing about 3 ml of RPMI medium (Sigma, St. Louis, MO). The sperm was released from epididymis in the medium, which was centrifuged at 2,000 × g for 15 min and the pellet was collected. The sperm pellet was washed one time with PBS and collected and stored until assayed for gene expression and methylation measurements.

### Cell Culture

Neural stem cells were prepared and differentiated to POMC/β-endorphin neurons using cAMP and pituitary adenylate cyclase-activating polypeptide (PACAP) and maintained in cultures as we described previously (Sarkar et al., 2008). The HEK293FT cell line was purchased from Life Technologies (Carlsbad, CA) and was grown in DMEM (high glucose) medium with 10% fetal bovine serum (FBS), 0.1 mM MEM non-essential amino acids, 6mM L-glutamine, 1 mM sodium pyruvate, and 1% Pen-Strep (Life Technologies) in a 37°C incubator with 7.5% CO_2_.

### Real-Time PCR for Gene Expression Measurements

Gene expression levels of *Pomc and Sry* in rat MBH and sperm were measured by quantitative real-time polymerase chain reaction (RT-PCR) (SYBR Green assay). Total RNA from MBH and sperm were extracted using an RNeasy kit (Qiagen, Germantown, MD). Total RNA (1 μg) was converted to first-strand complementary DNA (cDNA) using a high-capacity cDNA reverse transcription kit (Life Technologies). The primer sequences used for *Pomc*, *Sry, Gapdh, 18S*, and *Rpl19* are given in Table 1. Real-time quantitative PCR was performed at 95°C for 5 min, followed by 40 cycles of 95°C for 15 s, 60°C for 30 s, and 72°C for 40 s in the Applied Biosystems 7,500 real-time PCR system (Foster City, CA). The quantity of target genes (*Pomc, Sry*) and the three reference genes (*Gapdh, 18S, Rpl19*) were measured using the standard curve method. Target-gene expression was normalized with reference gene expression levels.

**TABLE 1:** Primer sequences.

**Primer name**	**Sequence**
*Pomc* FP	5′-CAAGAGGGAGCTGGAAGGCGAGC-3′
*Pomc* RP	5′-TCACTGGCCCTTCTTGTG-3′
*Pomc* M FP	5′-GTTAGGTGTGCGTTTTAGC-3′
*Pomc* M RP	5′-CTAACAACGCTTCTACAACG-3′
*Pomc* UM FP	5′-GGGTTAGGTGTGTGTTTTAGT-3′
*Pomc* UM RP	5′-CCTAACAACACTTCTACAACACA-3′
*Sry* FP	5′-GCGCCCCATGAATGCAT-3′
*Sry* RP	5′-TGGGATTCTGTTGAGCCAACT-3′
*Sry* MFP	5′-GTTTGTGTTATTAAGTGTTTTGAAATT-3′
*Sry* MRP	5′-TTTTTTTTTATTTTTGTGTATAGGA-3′
*Sry* UMFP	5′-GTTTGTGTCATTAAGTGCTTTGAAACC-3′
*Sry* UMRP	5′-CTCTCCTTCATCTTTGTGCACAGGA-3′
*Pomc* prom chip FP	5′-ATAGTCTGAGGCTGGCAGGA-3′
*Pomc* prom chip RP	5′-GCATCAGATTTCCCCAGTGT-3′
*Pomc* prom FP	5′-AATCCTAGTCCCCCTGCCAG-3′
*Pomc* prom RP	5′-ACACCCTTACCTGTCGCG-3′
*Sry* BS1 mutant FP	5′-TGGGGATGGAGACAGACTGTTTTAACTCACTTGCAC ACACTCC-3′
*Sry* BS1 mutant RP	5′-GGAGTGTGTGCAAGTGAGTTAAAACAGTCTGTCTC CATCCCCA-3′
*Sry* BS2 mutant FP	5′-CGCGTGGCCGGGGATTCGCTAAATGCGTTG CAGAA-3′
*Sry* BS2 mutant RP	5′-TTCTGCAACGCATTTAGCGAATCCCCGGC CACGCG-3′
*Gapdh* FP	5′-AGACAGCCGCATCTTCTTGT-3′
*Gapdh* RP	5′-CTTGCCGTGGGTAGAGTCAT-3′
*18s* FP	5′-GTAACCCGTTGAACCCCATT-3′
*18s* RP	5′-CCATCCAATCGGTAGTAGCG-3′
*rpl-19* FP	5′-AATCGCCAATGCCAACTCTCG-3′
*rpl-19* RP	5′-TGCTCCATGAGAATCCGCTTG-3′
Control sense ODN2	5′-AGGGCCATGTCAAGCGCCCCAT-3′
Control sense ODN3	5′-TCTAGATAGCATGGAGGGCC-3′
Antisense ODN2	5′-CATGGGGCGCTTGACATGGCCC-3′
Antisense ODN3	5′-GGCCCTCCATGCTATCTAGA-3′

**FP, Forward primer; RP, reverse primer; MFP, methyl forward primer; MRP, methyl reverse primer; BS, binding site.**

### *In vitro* and *in vivo* Knockdown of Sry

Sense and antisense oligos (ODN2, ODN3) were used for *Sry* knockdown as it was described (Dewing et al., 2006). The sequences for the oligos were given in Table 1 and were designed using software from Exiqon. These oligos were purchased from Exiqon, and equal quantities were mixed in artificial cerebrospinal fluid at a final concentration of 1 μg/μl. For *in vitro* knockdown experiments, β-endorphin-producing neurons were seeded (25,000 cells/well) in a 12-well plate, and oligo 0.4 μg was added in 0.4 ml of medium in each well (final concentration of 1μg/ml medium) and incubated in a 37°C incubator with 7.5% CO_2_. The cells were harvested 48 h after transfection, and we pooled the cells from two wells in triplicate. Gene expression for *Sry* and *Pomc* was measured as it is described in section “Materials and Methods.” For *in vivo* knockdown experiments, oligos were injected into the third ventricle of the rats via intracerebroventricular infusion as we described earlier (Gangisetty et al., 2014). Male rats at the age of 90 days (*n* = 6) for each group were used for the experiment. Oligo mix prepared in artificial CSF (1 μg/μl) was injected 2 μl into each rat at the rate of 1 μl/min. Rats were allowed to recover, were sacrificed 72 h after oligo injection, and then brain tissue was collected. MBH was isolated from the brain and analyzed for *Sry* and *Pomc* gene expression as it was described in section “Materials and Methods.”

### Chromatin Immunoprecipitation (ChIP) Assay

ChIP assays were performed using a ChIP assay kit (Millipore Sigma, Saint Louis, MO) following instructions from the manufacturer. MBH collected from the brain tissue was placed on a small petri dish on ice and was minced into tiny pieces. Proteins were crosslinked with DNA from a tissue sample in 1% formaldehyde in PBS with protease inhibitors at room temperature for 30 min. The crosslinking was quenched with 0.125M glycine, and the sample was collected by centrifugation and washed twice with ice-cold PBS. The sample was suspended in SDS lysis buffer (with protease inhibitor) and homogenized to get uniform cell suspension. The crosslinked DNA was sheared into fragments the size of about 400–500 bp with a setting of 30% amplitude, with 15 s pulse on, 30 s pulse off for 10–12 cycles using the Qsonica ultrasonic cell disruptor (Qsonica, Newton, CT). The sample was centrifuged at 13,000 × g for 10 min, and we collected sonicated cell supernatant. The sample was diluted 10-fold in ChIP dilution buffer, and we used an aliquot for input. The remaining diluted sample was precleared with salmon sperm DNA/protein-A agarose. A ChIP assay was performed with a precleared sonicated sample with rabbit polyclonal SRY antibody (Thermo Fisher Scientific, Waltham, MA) (10 μg/sample) using a ChIP assay kit (Millipore Sigma, Burlington, MA) as specified in the manufacturer’s instructions. Equal concentration of the normal rabbit IgG was employed as negative control. The eluted ChIP samples and input sonicated samples were reverse crosslinked with 5M NaCl and incubated at 65°C overnight. DNA was extracted from these samples using a DNeasy micro kit (Qiagen). *Pomc* promotor-specific ChIP PCR primers (sequences in Table 1) were used to amplify the SRY enriched *Pomc* gene promoter region. The quantitative RT-PCR using SYBR Green assay was performed on the Applied Biosystems 7500 real-time PCR system. PCR conditions were 5 min at 95°C, followed by 40 cycles of 15 s at 95°C, 30 s at 60°C, and 40 s at 72°C. ChIP DNA sample’s quantity measures were normalized by their respective input sample’s DNA.

### Pomc Promoter Luciferase Assay

*Pomc* promoter region was PCR amplified from rat MBH genomic DNA using primers as listed in Table 1. The PCR product was cloned into basic PCR2.1 vector (Invitrogen, Carlsbad, CA), and the sequence was verified with M13R primer. The plasmid DNA was digested with Sac1 and Xho1 and cloned into PGL3 basic luciferase reporter vector (Promega, Madison, WI). The positive clones were sequence verified with RVP3, a sequencing primer for PGL3 vector. We constructed three different mutants with change in nucleotide sequences of *SRY* binding sites of the *Pomc* promoter. All three mutants were designed by altering *SRY* binding site 1 (mutant 1), binding site 2 (mutant 2), and 1 and 2 together (mutant 3, double mutant). We used PGL3 vector-containing wild-type *Pomc* promoter as a template to make these mutants using primers whose sequences are given in Table 1. A quick-change site-directed mutagenesis kit (Agilent Technologies, Santa Clara, CA) was used to make all mutants as per instructions from the manufacturer.

HEK293 cells were seeded 50,000 cells/well in a 96-well plate. The cells were transfected with 250 ng of reporter vector or vector-containing *Pomc* promoter wild-type or mutant plasmid DNA along with either *Sry* overexpression plasmid or empty vector (PCDNA3.0) using Lipofectamine 3000 reagent (Thermo Fisher Scientific). All cells were also transfected with a plasmid-carrying renilla luciferase for normalization purposes. After 24 h of transfection, we measured firefly luciferase and renilla luciferase activity using the Dual Glo luciferase assay system (Promega) in a BioTech model synergy HT plate reader (BioTech, Winooski, VT). Eight samples were used in each group in transfection studies. Luciferase activity was measured in duplicates.

### Statistical Analysis

Data were analyzed using Prism 5.0 (GraphPad Software). The data shown in the figures are mean ± standard error mean (SEM). The significant differences between different treatment groups were assessed with one-way analysis of variance (ANOVA) with *post hoc* analysis using the Newman-Keuls *post hoc* test. *P* < 0.05 was considered significant. The significant differences between two groups were analyzed using the unpaired *t*-test, and two-tailed *p*-value < 0.05 was considered significant.

## Results

### Fetal Alcohol Exposure Reduces *Pomc*, *Sry* Expression in the MBH and Sperm in F1 Offspring

**FIGURE 1:**
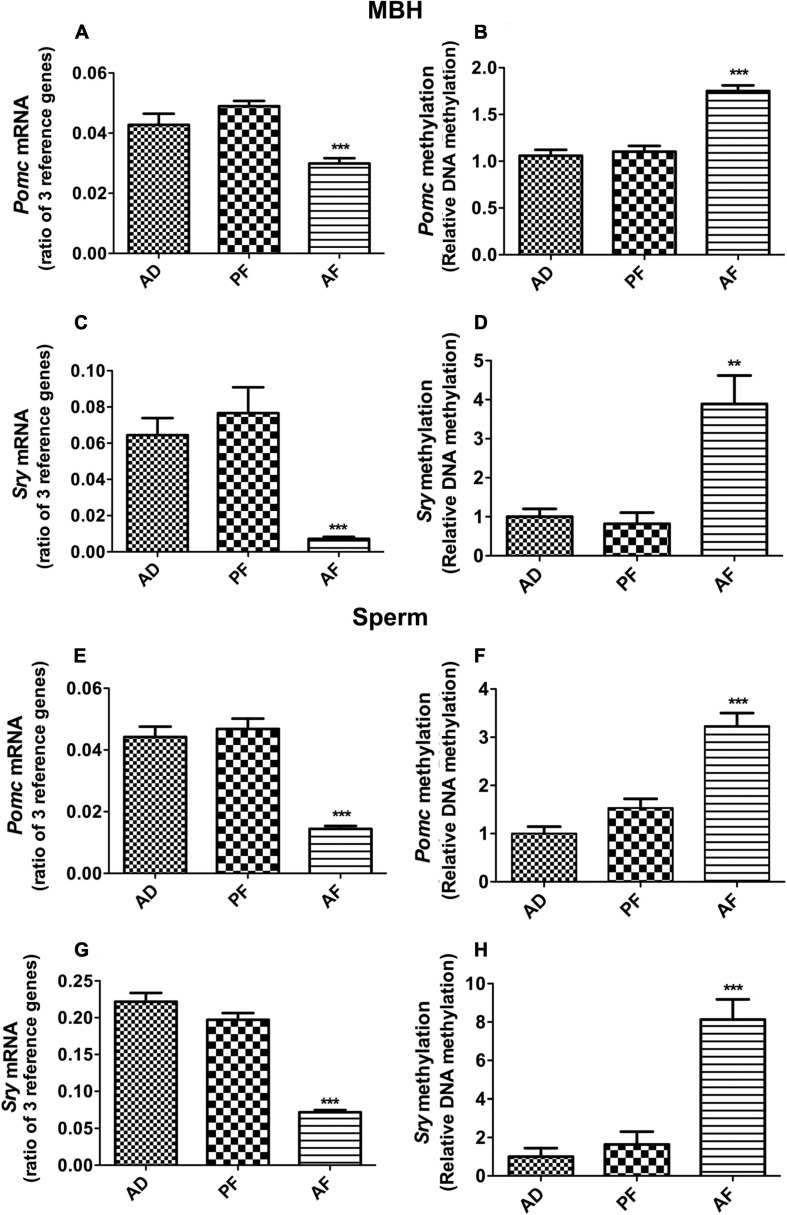
Effect of fetal alcohol exposure on the gene expression and promoter DNA methylation of *Pomc* and *Sry* genes in mediobasal hypothalamus (MBH) and sperm samples of F1 rat offspring. *Pomc* mRNA **(A,E)**, *Pomc* promoter DNA methylation **(B,F)**, *Sry* mRNA **(C,G)** and *Sry* promoter DNA methylation **(D,H)** levels in MBH and sperm samples of ad lib-fed (AD), pair-fed (PF), and alcohol-fed (AF) male rats from F1 generation offspring. Gene expression changes of *Pomc* and *Sry* genes were measured by quantitative RT PCR, and the quantities were normalized with ratios of three reference genes (*Gapdh, 18S, Rpl19*). Promoter DNA methylation of *Pomc* and *Sry* genes were measured by methylation-specific PCR, and methylation levels were measured as a ratio of methylated verses unmethylated DNA. Data are mean ± SEM (*n* = 6) and were analyzed using one-way ANOVA with the Newman-Keuls *post hoc* test; **p < 0.01, ***p < 0.001 between AF and controls (AD, PF).

We used a liquid diet model of alcohol feeding in pregnant rats between day 7 and 21 of pregnancy that is known to raise blood levels of alcohol in the range of 120–150 mg/dl (Miller, 1992) and produce offspring with endophenotypes similar to those found in human fetal alcohol spectrum disorders (e.g., high-anxiety behaviors, stress hyperresponsiveness, metabolic diseases; Ting and Lautt, 2006; Boyadjieva et al., 2009; Hellemans et al., 2010; Nash and Davies, 2017). We have also shown previously that FAE increases *Pomc* promoter DNA methylation and suppresses *Pomc* gene expression in the MBH and in sperm (Govorko et al., 2012). In this study, we confirmed that FAE increases *Pomc* DNA methylation and suppresses *Pomc* gene expression in the MBH and in sperm of Fischer 344 rats (Figures 1A,B,E,F). Furthermore, we show that FAE also increases *Sry* DNA methylation and suppresses *Sry* gene expression in the MBH and in sperm of Fischer 344 rats (Figures 1C,D,G,H). These data indicate that FAE results in a similar increase in DNA methylation and similar decrease in gene expression of both *Pomc and Sry* in sperm and the MBH of male offspring.

### Transgenerational Effect of Fetal Alcohol Exposure on *Sry* Expression in the MBH and Sperm

**FIGURE 2:**
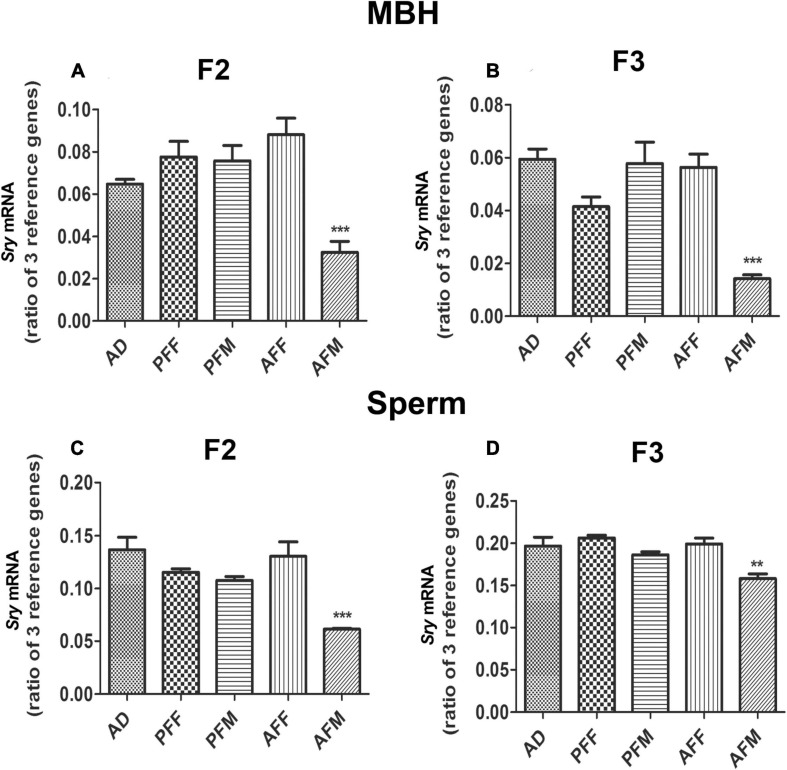
Transgenerational Effect of Fetal Alcohol Exposure on *Sry* Expression in MBH and Sperm Samples of Rat Offspring. *Sry* mRNA levels in MBH of F2 **(A)** and F3 **(B)** and in sperm of F2 **(C)** and of F3 **(D)** male rat offspring of ad lib-fed (AD), pair-fed male (PFM), pair-fed female (PFF), alcohol-fed male (AFM), or alcohol-fed female (AFF) rats. *Sry* mRNA levels were measured by quantitative RT PCR, and quantities were normalized using three reference genes (*Gapdh, 18S, Rpl19*). Data are mean ± SEM (*n* = 8) and were analyzed by one-way ANOVA with the Newman-Keuls *post hoc* test; **p < 0.01, ***p < 0.001 between AFM and controls (AD, PFF, PFM, AFF) in F2 and F3 generations.

Since FAE similarly affects the expression of *Pomc* and *Sry* genes in the F1 offspring, and FAE effects on *Pomc* gene expression have been shown to transmit for multiple generations (Govorko et al., 2012), we tested whether the FAE effect on *Sry* is also transmitted transgenerationally. We produced two different germlines, a male germline by breeding male fetal alcohol-exposed rats and their male offspring with normal females, and a female germline by breeding female fetal alcohol-exposed rats and their female offspring with normal males, as we have previously described (Govorko et al., 2012; Gangisetty et al., 2020). We also produced male (PFM) and female germlines (PFF) of control-fed rats or *ad libitum*-fed rats (AD). We measured the expression of *Sry* in F2 and F3 male offspring of male and female germlines to determine whether the fetal alcohol effect is transmitted through successive generations via germline. Like in the F1 generation, the F2 male progeny of male germlines (AFM) showed a significant reduction in *Sry* mRNA levels in the MBH (Figure 2A) and sperm (Figure 2C) as compared to the corresponding control groups. In the F3 generation, like in the F2 progeny, the F3 male progeny of the male germline (AFM) showed a reduction in *Sry* mRNA levels (Figures 2B,D). Other treatment groups did not differ from the control groups.

### *Sry* Knockdown Reduces Pomc Expression *in vitro* and *in vivo*

**FIGURE 3:**
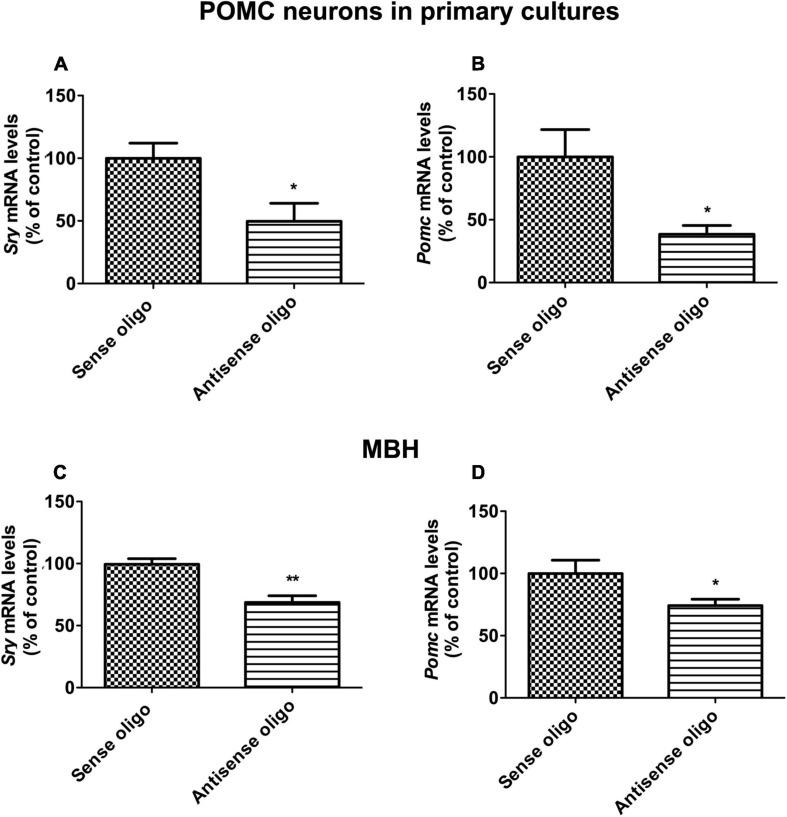
*Sry gene* knockdown effects on *POMC* expression *in vitro* and *in vivo*. *In situ* differentiated *POMC* neuronal cells from neuronal stem cells were transfected with antisense *Sry* oligo and sense control oligo. After 24 h of transfection, *Sry* mRNA **(A)** and *Pomc* mRNA **(B)** levels were measured by quantitative real-time PCR. *Sry* knockdown was performed *in vivo* through intracerebroventricular (icv) infusion of antisense *Sry* oligo and sense control oligo in male rats. After 72 h of infusion, *Sry* mRNA **(C)** and *Pomc* mRNA **(D)** levels were measured in MBH. Gene expression data was presented as percentage of sense control. Data are mean ± SEM (*n* = 6) and were analyzed using the unpaired *t*-test. *p < 0.05, **p < 0.01, between antisense oligo and sense oligo.

SRY has been shown to regulate the endocrine response to stress (Dickey et al., 2012). Hence the possibility is raised that the *Sry* gene might have a regulatory role in the Pomc gene control mechanism. To test this hypothesis, we first determined the effect of *Sry* gene knockdown, using antisense oligo specific to *Sry*, on *Pomc* gene expression in POMC/(β-endorphin neurons in cultures. The results show that antisense Sry oligo significantly reduced the levels of Sry mRNA as compared to those in the sense oligo-treated control. Additionally, the antisense *Sry* oligo treatment reduced the level of *Pomc* mRNA in these neurons as compared to those treated with control sense oligo (Figures 3A,B). We also tested the effect of *Sry* gene knockdown by antisense oligo *in vivo*, and results are shown in Figures 3C,D. We found that *Sry* antisense oligo effectively reduced *Sry* mRNA expression in the MBH (Figure 3C). Furthermore, *Sry* antisense oligo treatment reduced the *Pomc* mRNA level in the MBH as compared to those in sense oligo-treated controls (Figure 3D). These results indicate that *SRY* has a regulatory role in *Pomc* expression.

### FAE Reduces SRY Recruitment on to the *Pomc* Promoter

**FIGURE 4:**
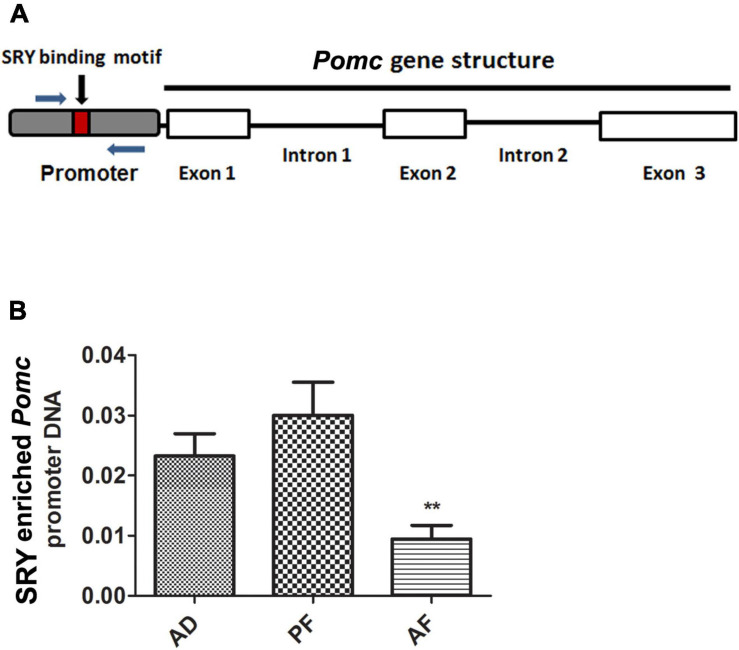
SRY recruitment onto the *Pomc* promoter in MBH of FAE rat offspring. A schematic diagram of *Pomc* gene structure comprising three exons and promoter. A small box within the promoter sequence represents the SRY binding motif. Arrows represent the ChIP primers spanning the *SRY* binding motif in *Pomc* promoter **(A).** SRY recruitment onto the *Pomc* promoter in MBH of AD, PF, and AF rat offspring were measured by ChIP assay. SRY-enriched *Pomc* promoter DNA was amplified by quantitative RT PCR using primers specific for *Pomc* promoter. SRY-enriched *Pomc* promoter DNA was normalized with input DNA **(B)**. Data are mean ± SEM (*n* = 6) and were analyzed using one-way ANOVA with the Newman-Keuls *post hoc* test; **p < 0.01 between AF and controls (AD, PF).

In order to understand how SRY regulates *Pomc* gene expression, we first evaluated *SRY* binding sites on the *Pomc* promoter using the TRANSFAC bioinformatics web tool which uses TRANSFAC version 8.3^1^ and then performed a ChIP assay using primers spanning the *SRY* binding site to identify its enrichment on the *Pomc* promoter (Figure 4A). The web tool predicted *SRY* binding sites within 1 kb of the promoter region of the *Pomc* gene. ChIP assay data revealed that SRY is enriched on the *Pomc* promoter in the MBH, and its enrichment is significantly reduced in AF offspring compared to AD and PF offspring (Figure 4B). These results suggest that FAE reduces SRY binding on the *Pomc* promoter in the MBH.

### SRY Directly Targets Pomc Promoter to Increase Its Expression

**FIGURE 5:**
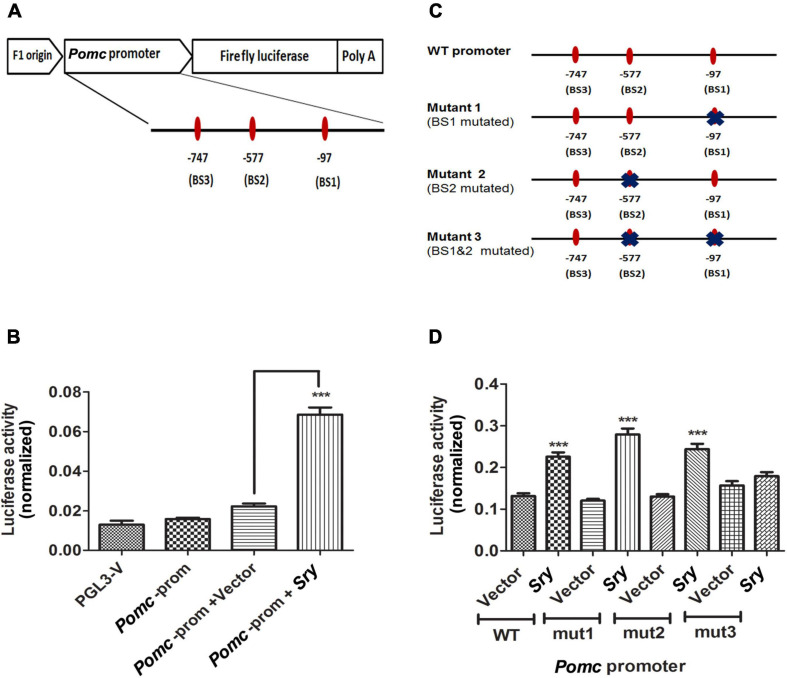
SRY directly targets its binding sites on *Pomc* promoter to regulate *Pomc* expression. A schematic representation of rat *Pomc* promoter was cloned upstream of firefly luciferase in a reporter luciferase vector. The three SRY binding sites (BS1, BS2, and BS3) are represented as ovals along with the horizontal line represented as the *POMC* promoter region. Each *SRY* binding site is indicated with its location relative to the exon 1 **(A)**. *Pomc* promoter luciferase activity is measured in HEK293 cells transfected with a reporter plasmid containing *Pomc* promoter along with *Sry* overexpression plasmid or vector alone. *Pomc* promoter activity is measured as firefly luciferase activity normalized with renilla luciferase **(B)**. *Pomc* promoter containing wild-type SRY binding sites and mutants with altered SRY binding sites represented in a schematic diagram were cloned in a reporter vector upstream of firefly luciferase, thereby generating 3 different mutants. Each mutation on the SRY binding site was marked with X on an oval shape representing the binding site **(C)**. *Pomc* promoter activity of HEK293 cells transfected with a reporter plasmid carrying *Pomc* promoter with *SRY* binding sites, wild-type or mutant1 (mut1) or mutant2 (mut2) or mutant3 (mut3), as represented in Figure 5C along with *SRY* overexpression plasmid or vector. *Pomc* promoter activity is measured as firefly luciferase activity normalized with renilla luciferase **(D)**. Data are mean ± SEM (*n* = 8) per group and were analyzed using one-way ANOVA with the Newman-Keuls *post hoc* test. ***p < 0.001 between SRY vs. vector.

In order to test that SRY may directly target *Pomc* promoter to increase its expression, we transfected a reporter plasmid carrying *Pomc* promoter along with a *Sry* overexpression plasmid or empty vector in HEK293 cells and measured luciferase activity. We analyzed the *Pomc* promoter region around 1 kb upstream of exon1 to detect *SRY* binding motifs using a bioinformatics web tool. We used a stringent threshold with > 95% sequence similarity and < 5% dissimilarity in the sequence to predict *SRY* binding sites on the *Pomc* promoter. It predicted three different *SRY* binding sites relative to exon 1 (−97, −577, and −747) in the *Pomc* promoter. We cloned the *Pomc* promoter upstream of firefly luciferase in a basic reporter luciferase vector (Figure 5A). Furthermore, we determined firefly luciferase activity as a measure of *Pomc* promoter and renilla luciferase activity for normalization purposes. We found that SRY significantly increased *Pomc* promoter luciferase activity compared to the vector control (Figure 5B). These results suggest that SRY regulate *Pomc* expression by acting on its binding motifs of *POMC* promoter.

We further confirmed the specificity of SRY binding onto its binding sites on *Pomc* promoter by mutating *SRY* binding sites using site-directed mutagenesis. We made three different mutant constructs by altering two of the *SRY* binding sites either singly or together, as shown in Figure 5C. We found that *SRY* increased *Pomc* promoter luciferase activity using the wild-type (WT) promoter sequence compared to vector. We also found that in mutant 1 (mut1: binding site1 mutated) and in mutant 2 (mut2: binding site2 mutated), *SRY* increased *Pomc* promoter luciferase activity similarly to that of the wild-type plasmid (Figure 5D). However, in mutant 3 (mut3: dual mutation of binding site1 and 2), *SRY* did not increase *Pomc* promoter activity compared to vector control, and it is in contrast to the wild-type promoter sequence (Figure 5D). Our results indicate that the mutation of a single binding site did not produce any effects, whereas the mutation of two binding sites significantly affected the SRY-induced *Pomc* promoter activity. Our results revealed that SRY may regulate *Pomc* gene expression by binding to two of its binding sites on the *Pomc* promoter.

## Discussion

In this study, we showed that FAE reduced *Sry* gene expression both in sperm and the MBH, and this effect on *Sry* gene expression persisted in F2 and F3 generations of male offspring that are derived from the male germline but not from the female germline. This transgenerational pattern of male germline transmission of fetal alcohol effects on *Sry* gene expression is similar to those seen in the fetal alcohol effect on *Pomc* gene expression (Govorko et al., 2012), suggesting that an interaction between these two genes might exist. The *Sry* gene on the Y chromosome is a master male sex-determining factor known to induce testis differentiation from bipotential gonads (Sinclair et al., 1990; Koopman et al., 1991). It is also responsible for generating male-specific properties of neuronal cells. SRY expression is localized in different regions of the brain, including the hypothalamus and substantia nigra of the midbrain regions that are involved in sex differentiation (Dewing et al., 2006). Studies in animal models have shown a possible effect of the specific part of the Y chromosome (Y^*NPAR*^) that comprises *SRY* on β-endorphin, a functional peptide of POMC (Botbol et al., 2011). This raises the possibility that SRY may participate in mediation of fetal alcohol effect on the *Pomc* gene in males.

We demonstrated in this study that *Sry* gene knockdown effectively reduced *Sry* mRNA expression, as well as reduced the Pomc mRNA level, in both β-endorphin neurons in cultures and in the rat MBH (where β-endorphin neurons are accumulated) *in vivo*. Additionally, ChIP assay data showed that SRY is enriched on the *Pomc* promoter in the MBH, and its enrichment is significantly reduced in fetal alcohol-exposed male offspring. Furthermore, we confirmed the specificity of SRY binding on *Pomc* promoter by mutating *SRY* binding sites using site-directed mutagenesis and showing that SRY binding on *Pomc* promoter is required for its action. These data suggest for the first time that SRY regulates *Pomc* gene expression by binding on the *Pomc* promoter.

The present data also identify the sites where SRY binds to *Pomc* promoter to enhance its activity. A bioinformatics web tool used with a stringent threshold of more than 95% sequence similarity predicted three SRY binding sites (1, 2, and 3) located (−97, −577, −747) upstream of exon1. We found that SRY-increased *Pomc* promoter activity was unchanged when binding sites 1 or 2 were mutated individually, but it was lost when both of these two sites were mutated simultaneously, indicating a requirement of SRY binding to sites 1 and 2 to promote *Pomc* gene expression. A similar mechanism has been reported in a study where SRY binding to two sites on the *Tcf21* promoter are shown to be required for *Tcf21* gene transcription during male gonadal sex determination (Bhandari et al., 2011).

Our data highlights the novel finding that FAE-induced alteration of *Sry* expression and promoter methylation correlate with *Pomc* in the hypothalamus and sperm. Other evidence from our study, such as *Sry* knockdown altering *Pomc* expression, SRY binding on *Pomc* promoter, and SRY-induced *Pomc* promoter activity, clearly suggests that SRY plays a role in the regulation of *Pomc*. However, our study did not conclude whether SRY mediates the FAE-induced male germline transmission of POMC expression changes in the hypothalamus for multiple generations. Further studies determining the role of SRY on FAE-modified *Pomc* transcription in F2 and F3 offspring are needed to establish the role of this transcription factor in the transgenerational effects of FAE.

The germline transmission of phenotypic and epigenetic traits was reported mostly in animal studies. Some human studies provided evidence for intergenerational transmission of risk variants with the paternal lineage (Liu et al., 2005; Zuo et al., 2013). Studies also showed a grandparent with a major depressive disorder with alcohol abuse greatly increased the risk of an individual having a major depressive disorder (Olino et al., 2008). Other studies also showed that grandmothers of children with fetal alcohol syndrome were found to have a much greater rate of history of alcohol abuse, suggesting a generational phenomenon in FASDs (Kvigne et al., 2008). These human studies support the concept of multigenerational and transgenerational inheritance of psychiatric disorders with alcohol drinking. However, more clinical studies are needed to examine FASD manifestations with paternal lineage and the mechanism behind this phenomenon.

In conclusion, the current study provide some insights to our understanding of the mechanism of fetal alcohol-induced male germline transmission. More studies are required to understand the exact mechanism by which SRY plays a critical role in transmitting fetal alcohol-induced changes of *Pomc* gene expression through the male germline.

## Data Availability Statement

All relevant data are included in this manuscript. Any requests for materials will be fulfilled upon request through appropriate means.

## Ethics Statement

All rat studies were performed with approved protocols in compliance with the Association for the Assessment and Accreditation of Laboratory Animal Care and Institutional Animal Care and Use Program, Rutgers, The State University of New Jersey Rutgers University.

## Author Contributions

DS designed the project and wrote the manuscript. OG helped in research design, participated in breeding and feeding the animals, contributed to sample collections, conducted biochemical assays, and helped in writing the manuscript. EM participated in conducting biochemical assays. All authors contributed to the article and approved the submitted version.

## Conflict of Interest

The authors declare that the research was conducted in the absence of any commercial or financial relationships that could be construed as a potential conflict of interest.

## Footnotes

1 http://alggen.lsi.upc.es/cgi-in/promo_v3/promo/promoinit.cgi?dirDB=TF_8.3

## Abbreviations

AD: fed *ad libitum* with rat chow
AF: fed with a liquid diet containing 6.7% alcohol
AFF: AF female
AFM: AF male
ANOVA: one-way analysis of variance
ChIP: chromatin immunoprecipitation
FAE: fetal alcohol exposure
FBS: fetal bovine serum
GD: gestational day
HPA: hypothalamic-pituitary-adrenal
MBH: mediobasal hypothalamus
PACAP: pituitary adenylate cyclase-activating polypeptide
PF: pair-fed with isocaloric liquid diet
PFF: PF female
PFM: PF male
POMC: proopiomelanocortin
RT-PCR: real-time polymerase chain reaction
SEM: standard error mean
SRY: sex-determining region Y.
